# Rethinking childhood-onset schizophrenia: neurodevelopmental comorbidities shape the psychopathological profile

**DOI:** 10.3389/fpsyt.2026.1918377

**Published:** 2026-10-12

**Authors:** Michelangelo Di Luzio, Maria Pontillo, Barbara D’Aiello, Giulia D’Amario, Ilaria Bertoncini, Roberto Averna, Milena Labonia, Stefano Vicari

**Affiliations:** 1 Child and Adolescent Neuropsychiatry Unit, Bambino Gesù Children’s Hospital, IRCCS, Rome, Italy; 2 Department of Neuroscience, Catholic University, Rome, Italy; 3 Life Sciences and Public Health Department, Catholic University, Rome, Italy

**Keywords:** childhood onset, early onset, neurodevelopment, psychopathology, schizophrenia

## Abstract

**Objectives:**

Childhood-onset schizophrenia (COS), defined by the onset of psychotic symptoms before the age of 13 years, is a rare and severe form of schizophrenia. Despite growing interest, little is known about how neurodevelopmental comorbidities shape its clinical presentation. This study aimed to examine whether COS with comorbid neurodevelopmental disorders differs in psychopathological profile from COS with psychiatric comorbidities and from COS without comorbidities.

**Methods:**

Sixty-one children diagnosed with COS and evaluated at symptom onset in a specialized early psychosis service were included and classified into three subgroups: COS with neurodevelopmental comorbidities, COS with psychiatric comorbidities, and COS without comorbidities. Psychopathological dimensions were assessed using the Kiddie Schedule for Affective Disorders and Schizophrenia for School-Aged Children – Present and Lifetime Version and the Structured Interview for Psychosis-Risk Syndromes and the Scale of Prodromal Symptoms (SIPS/SOPS). Group differences were examined using MANOVA and mixed-design repeated-measures ANOVA, repeated with IQ as a covariate, with False Discovery Rate correction across all SIPS measures. A secondary analysis separated intellectual disability from autism spectrum disorder and/or attention-deficit/hyperactivity disorder.

**Results:**

Children with COS and neurodevelopmental comorbidities showed an earlier age at onset, lower IQ, greater bizarre thinking and less social anhedonia. In contrast, COS with psychiatric comorbidity and COS without comorbidities exhibited largely overlapping profiles, marked by greater severity of negative symptoms and more organized thought processes. Within the neurodevelopmental group, the symptom pattern did not appear to be driven by specific diagnoses.

**Conclusions:**

These preliminary findings are consistent with variation in the configuration of psychopathological features within COS along a continuum, and highlight the relevance of neurodevelopmental trajectories in shaping symptom expression. Accounting for neurodevelopmental comorbidities may be crucial for improving early diagnostic characterization and informing targeted therapeutic strategies in pediatric schizophrenia.

## 1 Introduction

Schizophrenia is a severe psychiatric disorder characterized by positive symptoms (e.g., delusions and hallucinations), negative symptoms (e.g., blunted affect, asociality, avolition, alogia, and anhedonia), and disorganized symptoms (e.g., disorganized speech and behavior), as outlined in the DSM-5-TR (1).

Childhood-onset Schizophrenia (COS) is a distinct form of schizophrenia that appears at a very young age, typically before the age of 13 (2, 3). The diagnostic criteria for COS are shared with both adult-onset schizophrenia (AOS), which presents after the age of 18, and early-onset schizophrenia (EOS), where symptoms begin between 13 and 18 years (2–6). COS is exceptionally rare, and comprehensive epidemiological data are limited due to the limited research available (7). Nevertheless, available evidence indicates substantial continuity between COS and later-onset forms of schizophrenia in terms of core clinical features, genetic vulnerability, and neurobiological alterations, although these manifestations tend to be more severe in COS (2, 8, 9). It is widely accepted that COS presents a worse clinical profile, characterized not only by later and often less effective treatment (4), but also by more severe premorbid developmental impairments, more pronounced cognitive deficits and higher rates of neurodevelopmental comorbidities, such as Autism Spectrum Disorders (ASD) and Attention-Deficit/Hyperactivity Disorder (ADHD) compared to EOS and AOS (7, 10–14). From a psychopathological perspective, auditory hallucinations and negative symptoms are among the most prominent clinical features of COS, EOS and AOS, although few studies have specifically explored delusions and disorganization in COS (15, 16).

Interest in COS has increased in recent years; however, evidence on its clinical characteristics and developmental trajectories remains scarce and fragmented (17). Despite its severity and distinctiveness, the disorder is still supported by a limited empirical base, and a well-defined clinical profile has yet to be established. Even less is known about how this profile may vary in relation to neurodevelopmental comorbidities. In a previous study by our research group, this area was investigated but both COS and EOS patients were included in the study (18), leaving the specific contribution of neurodevelopmental alterations in COS yet to be clarified.

To date, no study has systematically isolated a COS subgroup with specific psychopathological features based on the presence of neurodevelopmental comorbidities. This lack of targeted investigation could represent a crucial gap in the understanding of COS heterogeneity, limiting the development of more tailored diagnostic and clinical approaches.

In the present study, we aim to address this gap by focusing exclusively on individuals with COS. Specifically, we seek to provide a comprehensive psychopathological characterization of COS and to examine whether and how the clinical presentation differs according to the presence of neurodevelopmental disorders. To this end, we compare COS subgroups defined by the presence of neurodevelopmental comorbidity, psychiatric comorbidities, or the absence of comorbid conditions.

## 2 Materials and methods

### 2.1 Participants

Participants were 70 help-seeking children consecutively admitted between 2022 and 2024 to the Child and Adolescent Neuropsychiatry Unit of Bambino Gesù Children’s Hospital (Rome, Italy), specifically to its specialized program for clinical high-risk states and very early-onset psychosis. Patients attending this specialized Day Hospital were referred by other clinicians following an initial evaluation, including the Emergency Department, the inpatient ward, as well as external secondary care units (e.g., child psychiatrists from national healthcare centers).

Children younger than 13 years were eligible if they met DSM-5-TR criteria for schizophrenia. Exclusion criteria were: (a) current or past brain injury or significant neurological or organic disorders; (b) diagnosis of substance- or medication-induced psychotic disorder, or psychotic disorder due to another medical condition; and (c) the concurrent presence of both a comorbid neurodevelopmental disorder and a comorbid psychiatric disorder. This latter criterion was applied to reduce diagnostic heterogeneity and to allow the identification of relatively homogeneous clinical subgroups. The aim of the study was not to estimate real-world comorbidity rates, but rather to identify psychopathological features associated with each comorbidity pattern, enhancing the interpretability of between-group comparisons.

Nine participants (12.8%) were excluded based on these criteria, resulting in a final sample of 61 children. No eligible participant declined participation. Descriptive characteristics of the nine participants excluded from the final sample are reported in **Supplementary Table 1** (**Supplementary Materials**). Given their limited number and the exploratory aim of the study, no formal inferential testing was performed on this subgroup.

The final sample was divided into three groups. Group 1 (G1) consisted of 20 children (32.8%) with COS and a comorbid neurodevelopmental disorder. Group 2 (G2) included 20 children (32.8%) with COS and a psychiatric comorbidity. Group 3 (G3) comprised 21 children (34.4%) with COS and no psychiatric or neurodevelopmental comorbidities. Group 2 was included to allow comparison between COS with neurodevelopmental disorders, COS without comorbidities, and COS with non-neurodevelopmental psychiatric comorbidities.

At the time of assessment, none of the participants was receiving regular pharmacological treatment.

All participants and their parents or legal guardians received detailed information about the study procedures and objectives. Written informed assent was obtained from all children, and written informed consent was provided by parents or legal guardians. The study was approved by the Ethics Committee of Bambino Gesù Children’s Hospital.

### 2.2 Clinical assessment

All participants underwent the standard clinical assessment protocol used at admission to the specialized clinical high-risk states and very early-onset psychosis service. Assessments were conducted by child and adolescent neuropsychiatrists and psychologists trained in the administration of structured and semi-structured diagnostic instruments and experienced in evaluating psychotic symptoms in pediatric populations, both with and without neurodevelopmental comorbidities.

A comprehensive clinical interview was conducted with both participants and their parents or caregivers. The presence of neurodevelopmental or psychiatric disorders predating the onset of psychotic symptoms was established through clinical evaluation and retrospective developmental history. Demographic and clinical information, including age, sex, family history of psychosis, previous or current pharmacological treatments, and substance use, was systematically collected.

Psychiatric and neurodevelopmental diagnoses were assessed using the Kiddie Schedule for Affective Disorders and Schizophrenia for School-Aged Children – Present and Lifetime Version (K-SADS-PL) (19), a semi-structured diagnostic interview designed to evaluate current and lifetime psychopathology according to DSM-5 criteria. The interview integrates information obtained from both the child and a parent or primary caregiver, enhancing diagnostic accuracy by combining self-reported experiences with caregiver observations of developmental and behavioral history.

General intellectual functioning was assessed using the Wechsler Intelligence Scale for Children – Fifth Edition (20). The total IQ score was used as an index of overall cognitive ability.

Psychotic symptomatology was evaluated using the Structured Interview for Psychosis-Risk Syndromes (SIPS) and the Scale of Prodromal Symptoms (SOPS) (21). The SIPS/SOPS assesses four symptom dimensions: positive symptoms, negative symptoms, disorganized symptoms, and general symptoms. Each item is rated on a 7-point scale ranging from 0 (absent) to 6 (extreme severity, or psychotic intensity in the case of positive symptom items). To avoid ambiguity, it should be specified that item N5, referred to as ideational richness, assesses a reduction in ideational richness; therefore, higher scores indicate poorer ideational content. The general symptoms dimension of the SIPS/SOPS, comprising sleep disturbance, dysphoric mood, motor disturbances, and impaired tolerance to normal stress, was not included in the present analyses. Although these symptoms contribute to the broader clinical characterization provided by the SIPS/SOPS, they were not included because the present study specifically focused on positive, negative, and disorganized symptom dimensions in characterizing the psychotic symptom profile.

Given the young age of participants and the presence of neurodevelopmental disorders in a subset of the sample, both the K-SADS-PL DSM-5 and SIPS/SOPS interviews were conducted using information obtained from children and caregivers. Consistent with procedures adopted in previous studies (22–25), interviews were administered face-to-face, with caregiver support when appropriate. Clinicians adapted the administration to each child’s developmental level by simplifying language, breaking down complex questions, providing concrete or visual examples, and conducting multiple shorter sessions when needed. For participants with limited expressive language or intellectual disability (ID), greater weight was given to behavioral observations and caregiver reports. In cases of discrepant reports, clinician-rated scores were assigned following the most conservative criterion. These adaptations preserved the original structure and scoring rules of the instruments to ensure developmental appropriateness without compromising diagnostic validity.

Global functioning across family, school, and social domains was assessed using the Children’s Global Assessment Scale (26), which yields scores ranging from 1 (severe impairment requiring constant supervision) to 100 (superior functioning).

### 2.3 Statistical analysis

Descriptive statistics were calculated for demographic and clinical variables. Multivariate analysis of variance (MANOVA) was used to examine sex differences in SIPS/SOPS symptom dimensions. A repeated-measures ANOVA (RM-ANOVA) was conducted to compare the severity of positive, negative, and disorganized symptoms across the whole sample.

Group differences among the three COS subgroups were examined using MANOVA, with the SIPS measures as dependent variables and group as the between-subjects factor. Pillai’s Trace and Wilks’ Lambda were reported as multivariate test statistics. To further examine whether the pattern of symptom expression across the individual SIPS measures differed among groups, a mixed-design RM-ANOVA was conducted, with group as the between-subjects factor and SIPS measure as the within-subjects factor, applying Greenhouse–Geisser correction for violations of sphericity. Given the significant group difference in IQ, both models were repeated with IQ as a covariate, followed by univariate analyses of individual SIPS measures before and after adjustment.

To address multiple comparisons, the Benjamini–Hochberg False Discovery Rate (FDR) procedure was applied across all measures of SIPS reported in **Table 1** (18 comparisons), separately for the unadjusted and IQ-adjusted models. Only effects surviving FDR correction were considered robust, whereas nominally significant effects that did not survive correction were considered exploratory. As a secondary exploratory analysis, the neurodevelopmental group was subdivided into participants with ID and those with ASD or ADHD, and the comparisons were repeated using the same analytical procedure.

**Table 1:** SIPS items in the total sample and comparisons across the three COS groups.

Variables	Total	G1	G2	G3	F	P-value	η²p
SIPS positive items							
P1: Unusual Thought Content/Delusional Ideas	4.84 (1.90)	4.30 (2.20)	5.55 (1.14)	4.66 (2.05)	2.37	0.102	0.07
P2: Suspiciousness/Persecutory Ideas	3.90 (2.20)	3.70 (2.31)	3.90 (2.44)	4.09 (1.92)	0.160	0.852	0.005
P3: Grandiose Ideas	1.56 (1.26)	1.55 (1.43)	1.45 (1.27)	1.66 (1.11)	0.148	0.863	0.005
P4: Perceptual Abnormalities/Hallucinations	4.11 (2.35)	4.80 (2.14)	3.60 (2.50)	3.95 (2.35)	1.39	0.256	0.046
P5: Disorganized Communication	2.56 (2.07)	2.75 (2.38)	2.60 (2.16)	2.33 (2.37)	0.208	0.813	0.007
P total	16.97 (4.81)	17.10 (5.17)	17.10 (4.88)	16.71 (4.60)	0.04	0.958	0.001
SIPS negative items							
**N1: Social Anhedonia**	5.23(1.45)	**4.25(1.61)**	**5.70 (1.12)**	**5.71 (1.10)**	8.42	0.001*	0.225
N2: Avolition	4.98 (1.64)	4.70 (1.92)	5.60 (0.94)	4.66 (1.77)	2.19	0.12	0.070
N3: Expression of Emotion	5.13 (1.43)	4.95 (1.60)	5.45 (0.94)	5.00 (1.64)	0.73	0.483	0.025
N4: Experience of Emotions and Self	5.08 (1.45)	4.95 (1.57)	5.45 (0.88)	4.86 (1.74)	0.97	0.383	0.033
**N5: Ideational Richness**	4.59 (1.74)	**3.90 (2.02)**	**5.25 (1.33)**	4.62 (1.59)	3.25	**0.046^†^**	0.101
N6: Occupational Functioning	4.75 (1.39)	4.55 (1.63)	5.15 (1.08)	4.57 (1.39)	1.20	0.308	0.040
**N total**	29.77 (6.73)	**27.30 (7.01)**	**32.60 (3.83)**	29.42 (7.80)	3.39	**0.040^†^**	0.105
SIPS disorganized items							
D1: Odd Behavior or Appearance	5.15 (1.49)	5.65 (0.93)	4.85 (1.53)	4.95 (1.80)	1.75	0.182	0.057
**D2: Bizarre Thinking**	4.74 (1.61)	**5.75 (0.44)**	**4.40 (1.39)**	**4.09 (2.04)**	7.33	0.001*	0.202
D3: Trouble with Focus and Attention	4.31 (1.98)	4.65 (1.69)	4.10 (2.02)	4.19 (2.22)	0.43	0.648	0.015
D4: Impairment in Personal Hygiene	3.23 (1.86)	3.60 (1.95)	2.65 (1.59)	3.43 (1.96)	1.50	0.231	0.049
D total	17.43 (5.43)	19.65 (3.54)	16.00 (4.69)	16.66 (6.95)	2.71	0.075	0.085

G1, Group 1; G2, Group 2; G3, Group 3; SIPS, Structured Interview for Psychosis-Risk Syndromes.

Values are reported as mean (SD);.

* p ≤ 0.05, significant after Benjamini–Hochberg FDR correction across the full family of 18 SIPS comparisons;.

† p ≤ 0.05, significant at the uncorrected level only; did not survive FDR correction and should be considered exploratory;.

*Post hoc* pairwise comparisons: Social anhedonia (N1), G1 < G2 (p = 0.003), G1 < G3 (p = 0.002); Bizarre thinking (D2), G1 > G2 (p = 0.015), G1 > G3 (p = 0.002); Ideational richness (N5), G1 < G2 (p = 0.040); Negative total, G1 < G2 (p = 0.037).

Bold values were found to be significant in the analysis.

*Post hoc* comparisons were conducted using Bonferroni-adjusted confidence intervals (95%). Associations between categorical variables (group, sex, family history of psychosis) were examined using chi-square tests. Statistical analyses were performed using SPSS Statistics, version 22 (IBM Corp., Armonk, NY, USA). A two-tailed p value ≤ 0.05 was considered statistically significant.

## 3 Results

### 3.1 Sociodemographic and clinical characteristics of the total sample

The sociodemographic and clinical characteristics of the total sample are summarized in **Table 2**. The sample was predominantly male (57.4%), with a mean age at onset of 11.13 years (SD = 1.43) and a mean full-scale IQ of 92.16 (SD = 20.24), within the average range. Global functioning was markedly impaired (C-GAS = 44.08, SD = 6.73), while a family history of psychosis was uncommon, reported in 3.3% of first-degree and 4.9% of second-degree relatives.

**Table 2:** Sociodemographic and clinical characteristics of the total sample.

Variables	N (%) or mean (SD)
Male sex	35 (57.4%)
Age at onset	11.13 (± 1.43)
IQ	92.16 (± 20.24)
C-GAS	44.08 (± 6.73)
Family history of psychosis in first-degree relatives	2 (3.3%)
Family history of psychosis in second-degree relatives	3 (4.9%)

IQ, intelligence quotient; C-GAS, Children’s Global Assessment Scale.

Values are reported as N (%) or mean (SD). Percentages refer to the proportion of participants in the total sample.

### 3.2 Distribution of symptom dimensions in the total sample

Across the whole sample (**Table 1**), analysis of psychotic symptom severity revealed a significant effect of symptom dimension on SIPS/SOPS total scores (F_2,120_ = 95.64, p < 0.0001; η^2p^= 0.61). *Post hoc* Bonferroni comparisons indicated that negative symptoms (N total) were significantly more severe than both disorganized (D total; p < 0.0001) and positive symptoms (P total; p < 0.0001), whereas no difference was observed between positive and disorganized symptom totals (p = 0.91).

### 3.3 Sex differences across the total sample

No significant sex differences were observed for any positive or negative symptom dimensions or their total (all p > 0.05; **Supplementary Table 2**, **Supplementary Material**). In contrast, males showed higher scores than females on bizarre thinking [F _(1, 59)_ = 5.58, p = 0.021] and trouble with focus and attention [F _(1, 59)_ = 10.58, p = 0.002] as well as on the total disorganized score [F_(1, 59)_ = 8.61, p = 0.005]. No significant sex differences were observed for odd behavior or appearance (p = 0.08) or impairment in personal hygiene (p = 0.129).

### 3.4 Comparison between COS groups

#### 3.4.1 Sociodemographic and clinical comparisons across COS Groups

As shown in **Table 3**, the three groups did not differ in sex distribution, diagnostic categories, or family history of psychosis (all p > 0.05). Significant differences emerged for age [F _(2, 58)_ = 5.92, p = 0.005]. *Post hoc* Bonferroni analyses indicated that children in Group 1 were significantly younger than those in Group 2 (p = 0.024) and Group 3 (p = 0.007), whereas no difference emerged between Groups 2 and 3 (p = 1.00). IQ significantly differed between Groups [F _(2, 58)_ = 6.21, p = 0.004]. Group 1 showed significantly lower IQ scores than Group 2 (p = 0.008) and Group 3 (p < 0.001), while no difference was observed between Groups 2 and 3 (p = 1.00). Global functioning (C-GAS) did not differ across groups [F _(2, 58)_ = 0.38, p = 0.68].

**Table 3:** Comparison of sociodemographic and clinical characteristics across the three groups.

Variables	Group 1 N (%) or Mean (SD)	Group 2 N (%) or Mean (SD)	Group 3 N (%) or Mean (SD)	Test statistic (p-value)
Male sex	14 (23%)	9 (14.8%)	12 (19.7%)	*χ ^2^*_(2, 58)_ = 2.55 p = 0.27
**Age at onset**	**10.30 (1.62)**	**11.45 (0.82)**	**11.62 (1.39)**	*F*_(2, 58)_ *= 5.92* ***p = 0.005***
**IQ**	**80.10 (19.26)**	**98.55 (19.49)**	**97.57 (17.29)**	*F*_(2, 58)_ *= 6.21* ***p = 0.004***
C-GAS	44.05 (8.61)	45.05 (4.08)	43.19 (6.46)	*F*_(2, 58)_ *= 0.38* *p = 0.68*
First-degree relative	2 (3.3%)	0	0	*χ ^2^*_(2, 58)_ = 2.14 *p* = 0.34
Second-degree relative	1 (1.6%)	0	2 (3.3%)	*χ ^2^*_(2, 58)_ = 1.01 *p* = 0.60

IQ, intelligence quotient; C-GAS, Children’s Global Assessment Scale.

Values are reported as N (%) or mean (SD). Percentages refer to the proportion of participants in the total sample.

Bold values were found to be significant in the analysis.

The distribution of comorbid diagnoses across subgroups is detailed in **Table 4**. In Group 1, the most frequent neurodevelopmental comorbidity was ID (n = 12), followed by ASD (n = 4) and ADHD (n = 4). In Group 2, obsessive-compulsive disorder was the most common psychiatric comorbidity (n = 11), followed by generalized anxiety disorder (n = 6) and eating disorders (n = 3). By definition, Group 3 comprised participants without any comorbid diagnosis.

**Table 4:** Distribution of comorbidities across the three groups.

Comorbidity	Group 1 N (%) or Mean (SD)	Group 2 N (%) or Mean (SD)	Group 3 N (%) or Mean (SD)
No comorbidity	0	0	21 (32.79%)
Mood Disorder	0	2 (3.28%)	0
Generalized anxiety Disorder	0	6 (9.83%)	0
Obsessive Compulsive Disorder	0	11 (18.03%)	0
Eating Disorder	0	3 (4.91%)	0
PTSD	0	1 (1.63%)	0
Adjustment Disorder	0	1 (1.63%)	0
Autism Spectrum Disorder	4 (6.55%)	0	0
Intellectual Disability	12 (19.67%)	0	0
Learning Disorder	1 (1.63%)	0	0
ADHD	4 (6.55%)	0	0
Multicomplex Developmental Disorder	1 (1.63%)	0	0

PTSD, Post-traumatic Stress Disorder; ADHD, Attention Deficit and Hyperactivity Disorder.

Values are reported as N (%) or Mean (SD). Percentages refer to the proportion of participants in the total sample.

#### 3.4.2 Comparison of SIPS/SOPS items across COS groups

Significant group differences in psychotic symptoms are reported in **Table 1**.

The multivariate effect of group was not significant (Pillai’s Trace = 0.620, F_(30,90)_ = 1.34, p = 0.142; Wilks’ Lambda = 0.461, F _(30,88)_ = 1.38, p = 0.121). A separate mixed-design RM-ANOVA revealed a significant Group × SIPS measure interaction after Greenhouse–Geisser correction (F_(7.71, 223.57)_ = 2.45, p = 0.016), indicating that the pattern of symptom expression across the 18 SIPS measures differed among the three groups. After adjustment for IQ using MANCOVA, the multivariate effect of group remained non-significant (Pillai’s Trace = 0.537, F _(30,88)_ = 1.077, p = 0.383; Wilks’ Lambda = 0.529, F _(30,86)_, p = 0.385), and IQ was not significantly associated with the overall SIPS profile (Pillai’s Trace = 0.151, F _(15,43),_ p = 0.921). The Group × SIPS measure interaction was no longer significant after Greenhouse–Geisser correction (F_(7.79, 222.04)_ = 1.57, p = 0.138).

Follow-up univariate analyses were conducted to characterize group differences in individual SIPS measures, both before and after adjustment for IQ.

Positive Symptoms: No significant group differences emerged for any individual positive symptom item or for the total positive symptom score (all p > 0.05; **Table 1**).

Negative Symptoms: Nominally, significant group differences were observed for social anhedonia and ideational richness (**Table 1**). Both effects remained significant in the IQ-adjusted analyses (social anhedonia, F_(2,57)_ = 5.83, p = 0.002; ideational richness, F_(2,57)_ = 3.30, p = 0.027; **Supplementary Table 3**, **Supplementary Material**). *Post hoc* comparisons showed that Group 1 had lower social anhedonia scores than both Group 2 and Group 3, and lower ideational richness scores than Group 2, with no significant difference between Groups 2 and 3 (**Table 1**). No significant differences emerged for avolition, expression of emotion, experience of emotions and self, or occupational functioning (all p > 0.05). A significant group effect was also observed for the total negative symptom score, with lower scores in Group 1 than Group 2; this effect remained significant after adjustment for IQ (F_(2,57)_ = 2.91, p = 0.042).

Disorganized Symptoms: A significant group difference was observed for bizarre thinking, which remained significant after adjustment for IQ (F_(2,57)_ = 4.84, p = 0.005). Group 1 scored higher than both Group 2 and Group 3, with no significant difference between Groups 2 and 3 (**Table 1**). No significant group differences emerged for odd behavior or appearance, trouble with focus and attention, impairment in personal hygiene, or the total disorganized symptom score (all p > 0.05; **Table 1**).

After Benjamini–Hochberg FDR correction across the 18 SIPS measures, only social anhedonia and bizarre thinking survived in both models (unadjusted adjusted p = 0.009 for both; IQ-adjusted p = 0.036 and 0.045, respectively), whereas ideational richness (N5) and total negative symptoms (N total) did not. Thus, group differences in social anhedonia and bizarre thinking were robust to both multiple-testing correction and IQ adjustment, whereas the remaining nominal effects should be considered exploratory.

#### 3.4.3 Symptom profiles within the neurodevelopmental group: ID versus ASD/ADHD

To test whether these findings were primarily driven by ID, the neurodevelopmental group was split into ID (n = 12) and ASD/ADHD (n = 8), and comparisons were repeated with IQ as a covariate. The multivariate effect of symptom measures was significant (Pillai’s Trace = 0.998, F_(15,4)_ = 117.49, p < 0.001), whereas the symptom measures × group interaction was not (Pillai’s Trace = 0.904, F_(15,4)_ = 2.52, p = 0.193). After adjustment for IQ, the multivariate effect of symptom measures was not significant (Pillai’s Trace = 0.959, F_(15,3)_ = 4.65, p = 0.116), and neither the interactions with IQ (Pillai’s Trace = 0.676, F_(15,3)_ = 0.42, p = 0.891) nor group (Pillai’s Trace = 0.922, F_(15,3)_ = 2.35, p = 0.262) reached significance. In the repeated-measures analysis, the main effect of symptom measures remained significant after Greenhouse–Geisser correction (F_(3.58, 64.35)_ = 132.70, p < 0.001), whereas the symptom measures × group interaction did not (F_(3.58, 64.35)_ = 0.77, p = 0.538). The same held after IQ adjustment, with a significant main effect (F_(3.55, 60.27)_ = 3.16, p = 0.024) and non-significant interactions with IQ (F_(3.55, 60.27)_ = 0.35, p = 0.823) and group (F_(3.55, 60.27)_ = 0.52, p = 0.704). Overall, the pattern of SIPS symptom measures did not differ significantly between the ID and ASD/ADHD subgroups and was not moderated by IQ.

## 4 Discussion

### 4.1 Clinical features of the total COS sample

The present study investigated the psychopathological presentation of COS and its variation according to the presence of a co-occurring neurodevelopmental disorder. Consistent with previous literature, children with COS in our sample exhibited a severe clinical picture characterized by the co-occurrence of positive, negative, and disorganized symptoms within a context of markedly impaired global functioning (27). Notably, negative symptoms emerged as the most prominent dimension across the whole sample, showing higher total severity compared with both positive and disorganized symptoms. This finding underscores the central role of negative symptoms in COS psychopathology and aligns with evidence from EOS and AOS, in which negative symptoms are strongly associated with poorer premorbid functioning, reduced symptom remission, and worse long-term outcomes (28–30). In pediatric populations, negative symptoms—particularly social anhedonia, avolition, and affective blunting—may represent early markers of psychotic illness and are associated with greater functional impairment and poorer prognosis in both COS and EOS (31, 32).

The clinical presentation of positive symptoms was largely similar across groups. Hallucinations were consistently reported regardless of comorbidity status, while persecutory delusions were less frequently observed. In EOS and AOS at their first psychotic episode, persecutory delusions are usually more prevalent (33–35). This pattern may reflect developmental factors, as younger patients tend to present less systematized and less clearly articulated delusional content than adolescents or adults (33, 36).

Disorganized symptoms were overall less severe at the group level; however, specific manifestations such as bizarre thinking and behavioral disorganization were nonetheless clinically relevant. The relatively limited impact of disorganization on functional domains such as hygiene or role functioning may partly reflect the buffering role of caregivers during childhood. Disorganization is a poorly explored dimension of schizophrenia despite its central importance in the disorder (37–39). It is typically associated with poorer social functioning and worse prognosis (39, 40). Notably, the greater impact on social skills and metacognition appears to be more significant than that of positive symptoms (28, 41, 42).

Regarding global functioning, it is noteworthy that the C-GAS scores in our sample are very low, yet they do not differ across subgroups. This suggests that COS is a severe disorder irrespective of comorbidity.

### 4.2 Differing psychopathological profiles along the COS spectrum

Beyond the general characterization of COS, the present study explored whether psychopathological features vary along a common continuum in relation to different comorbidity patterns. The overall multivariate effect of group did not reach statistical significance providing no evidence of an overall difference in symptom severity across the three groups. What differed was the internal configuration of symptom dimensions, as indicated by the significant group × symptom dimension interaction. This interaction was attenuated when IQ was entered as a covariate; notably, however, IQ itself was not associated with the SIPS profile, and this attenuation should not be interpreted as indicating that intellectual functioning explains the observed pattern. At the level of individual symptom dimensions, two effects differentiated the groups robustly, remaining significant after both FDR correction for multiple comparisons and adjustment for IQ: social anhedonia and bizarre thinking. Taken together, these findings are consistent with variation in the relative expression of specific psychopathological dimensions within COS, rather than supporting the identification of categorically distinct clinical subgroups.

Children with COS and neurodevelopmental comorbidities (Group 1) showed an earlier age at onset, lower IQ, and a clinical profile marked by higher levels of bizarre thinking and lower levels of social anhedonia. Two further differences emerged at an uncorrected level and are worth describing, as they point in the same direction as the robust findings. Children in Group 1 showed lower ideational richness than those in Group 2 and also obtained lower total negative symptom scores than Group 2. Taken together, these effects would suggest that the attenuation of negative symptomatology in children with neurodevelopmental comorbidities is not confined to social anhedonia but extends to the negative dimension as a whole. However, neither difference survived FDR correction across the full set of SIPS measures. These findings should therefore be regarded as exploratory and interpreted with caution pending replication in larger samples.

Conversely, children with COS and psychiatric comorbidities (Group 2) and those without comorbidities (Group 3) showed largely overlapping psychopathological profiles, characterized by more organized thought processes and greater severity of social anhedonia (**Table 5**). The convergence of these two groups suggests that the presence of a neurodevelopmental disorder, rather than comorbidity per se, may be associated with variation in the configuration of COS psychopathology. This pattern is consistent with previous findings indicating that COS and EOS patients with neurodevelopmental comorbidities tend to present earlier onset and a relative predominance of positive and disorganized symptoms over negative symptoms (18). The association between male sex, neurodevelopmental comorbidities, and earlier age at onset reported in the literature (43) was not confirmed. However, male sex does appear to be more strongly associated with disorganization symptoms than female sex, which may suggest an indirect correlation between male sex and the Group 1 profile.

**Table 5:** Psychopathological profiles across the three COS groups.

	Group 1	Group 3	Group 2
Sex	M≅F	M=F	M=F
Age at onset	**earlier**	**later**	**later**
IQ	**lower**	**higher**	**higher**
Functioning	low	low	low
SIPS positive symptoms	commonly present	commonly present	commonly present
SIPS negative symptoms	**lower** (social anhedonia^a^, ideational richness^b^, total^b^)	**higher** (social anhedonia^a^)	**higher** (social anhedonia^a^, ideational richness^b^, total^b^)
SIPS disorganized symptoms	**higher** (bizarre thinking^a^)	**lower** (bizarre thinking^a^)	**lower** (bizarre thinking^a^)

Perimeter red line: Group 1 profile; perimeter blue line: Group 2-Group 3 profile;.

^a^Difference significant after FDR correction and adjustment for IQ; ^b^Nominally significant difference that did not survive correction for multiple comparisons; to be considered exploratory.

IQ, intelligence quotient; SIPS: Structured Interview for Psychosis-Risk Syndromes.

Bold values were found to be significant in the analysis.

A recognized challenge in schizophrenia research concerns the phenomenological overlap between negative symptoms and depressive or anxious symptomatology, which may inflate negative-symptom ratings in patients with comorbid mood or anxiety disorders. In the present sample, however, this overlap is unlikely to account for the observed pattern. Group 2 and Group 3 did not differ significantly on any negative-symptom dimension, including social anhedonia, despite Group 3 being, by definition, free of any anxious or depressive comorbidity. The finding that a comorbidity-free group displayed negative-symptom levels comparable to those of the psychiatric-comorbidity group argues against a confounding contribution of depressive or anxious symptoms and supports the interpretation of these features as intrinsic to COS psychopathology. Moreover, assessments were conducted in a specialized early-psychosis service using an instrument designed to rate psychotic and prodromal symptomatology (SIPS/SOPS), by clinicians trained to differentiate negative symptoms from overlapping affective phenomena.

A further consideration concerns whether the elevated bizarre thinking observed in children with neurodevelopmental comorbidities might reflect neurodevelopmental related differences in thinking or communication rather than psychotic disorganization proper — a well-recognized differential-diagnostic challenge. In the present study, however, bizarre thinking was not assessed in isolation: it was rated within an established DSM-5-TR diagnosis of schizophrenia, in children who all presented frank positive symptoms, including hallucinations. Disorganized features were therefore evaluated against a background of unequivocal psychotic symptomatology, rather than as stand-alone atypicalities in otherwise non-psychotic neurodivergent children. Moreover, as detailed below, the symptom pattern did not differ within the neurodevelopmental group according to the specific comorbid diagnosis: were bizarre thinking primarily an expression of neurodevelopmental communication differences, a greater contribution of ASD would have been expected, given that such features are most characteristic of autism. While the size of these subgroups renders this observation hypothesis-generating rather than conclusive, it does not support an interpretation of bizarre thinking as a mere reflection of neurodevelopmental communication style. However, rather than viewing neurodevelopmental features and psychotic disorganization as competing explanations, our findings suggest that in COS the neurodevelopmental substrate may shape the phenomenological expression of psychosis, with bizarreness of thought becoming more prominent when neurodevelopmental conditions co-occur. From a clinical standpoint, drawing a sharp boundary between the contribution of the neurodevelopmental condition and that of the psychotic process may be neither fully feasible nor necessarily informative, as the two dimensions are intrinsically intertwined in this population.

### 4.3 The contribution of intellectual disability

Because ID was the most frequent neurodevelopmental comorbidity in our sample, a sensitivity analysis was performed separating children with ID from those with ASD or ADHD. The overall pattern of symptom expression did not differ significantly between these two subgroups, either before or after adjustment for IQ. This suggests that the profile observed in children with neurodevelopmental comorbidity is not attributable to intellectual disability alone, but may represent a more general correlate of neurodevelopmental comorbidity in COS.

Within a dimensional approach, it is important to emphasize that low intellectual functioning and cognitive decline are not merely imported into the sample by the comorbidity: they are central features of COS, as also observed in AOS (44). A large body of literature has emphasized the importance of neurocognitive deficits in schizophrenia as an indicator of severity and poorer prognosis (28, 41, 42), and neurocognitive deficits and thought disorganization share overlapping neurobiological and functional substrates, particularly involving executive dysfunction and impaired integrative processing (40). Schizophrenia is associated with a decline in IQ, particularly at onset but also in the months preceding the emergence of psychosis (45), and patients with an earlier onset tend to exhibit more pronounced cognitive impairments (46).

Seen in this light, the association between neurodevelopmental conditions and disorganization observed in our sample is not easily separable from the disorder itself: in a condition whose earliest onset is characteristically accompanied by cognitive impairment, low intellectual functioning and disorganized thinking may represent converging expressions of a shared neurodevelopmental vulnerability rather than two independent contributions to be disentangled. For this reason, in our view excluding patients with ID from COS analyses is inadvisable. Applying an IQ cut-off of 70 would exclude not only COS patients with ID as a comorbidity, but also those whose disorder is itself characterized by substantial cognitive impairment — that is, potentially the most severe end of the COS spectrum. It should nonetheless be noted that the mean IQ of the neurodevelopmental group indicates that patients with moderate to severe intellectual disability were not represented in our sample.

### 4.4 A dimensional perspective

These findings are consistent with a dimensional view of schizophrenia psychopathology, in which the relative prominence of negative versus disorganized symptoms varies across the psychopathological continuum shaped by developmental pathways, rather than representing a uniform disease entity. Bleuler (37) already regarded positive symptoms as secondary to fundamental cognitive and behavioral alterations, such as withdrawal or loss of thought associations. Within this framework, the profile associated with neurodevelopmental comorbidity can be interpreted as reflecting the influence of neurodevelopmental alterations on the relative prominence of negative and disorganized symptoms, rather than as a distinct subtype or a separate diagnostic category.

From a clinical perspective, these differences may inform personalized intervention strategies. While pharmacological treatments primarily target positive symptoms, negative and disorganized symptoms remain largely resistant to medication. Psychosocial interventions focusing on neurocognition, metacognition, and social cognition may therefore be particularly relevant. Early identification of these profiles could facilitate tailored treatment planning and potentially improve long-term outcomes, although this hypothesis requires confirmation in prospective studies.

## 5 Limitations and strengths

The main strength of this study is that, to the best of our knowledge, it is the first study conducted in Italy exclusively on a population with COS and the first study attempting to characterize different psychopathological profiles based on comorbidity in this population. However, several limitations should be acknowledged. First, the sample size is relatively small; however, given the extreme rarity of COS, the ability to recruit and characterize a clinically well-defined cohort represents a notable strength. Second, the assessment relied on instruments not specifically designed for young children or for individuals with neurodevelopmental disorders. However, this limitation reflects a broader challenge in the field, as validated assessment tools for psychosis-risk and psychotic symptoms in these populations remain scarce. In line with previous research, standardized adaptations were therefore applied, and caregiver involvement was systematically incorporated (18, 24, 25). Third, the low prevalence of family history of psychosis may reflect limitations in data collection. Familial psychopathology was not assessed using a standardized family-history protocol or through direct evaluation but rather relied on information collected during routine clinical assessments. As a result, psychiatric disorders among family members may have been incompletely documented, particularly in cases where diagnostic information was unavailable or uncertain, potentially leading to an underestimation of familial psychosis. Beyond incomplete ascertainment, the low observed rate may also reflect the clinical composition of the sample, in which a substantial proportion of children presented neurodevelopmental comorbidities; in such cases, illness liability may be less strongly associated with a classic familial-aggregation pattern. Fourth, the exclusion of patients with both psychiatric and neurodevelopmental comorbidities limits generalizability. This decision was intentional, as including these patients would have generated a highly heterogeneous group, potentially obscuring meaningful psychopathological differences. Fifth, several methodological constraints should be considered when interpreting the present results. The overall multivariate effect of group did not reach statistical significance, the group × symptom dimension interaction did not remain significant after adjustment for IQ, and the subgroup sizes were modest — particularly in the sensitivity analysis separating ID from ASD/ADHD, where limited power precludes any conclusion about equivalence between neurodevelopmental conditions. Although the differences in social anhedonia and bizarre thinking survived both FDR correction and IQ adjustment, the remaining nominal effects should be regarded as exploratory, and all findings require replication in larger, independent samples. Finally, and relatedly, the cross-sectional rather than longitudinal design may limit the reliability of the findings more generally. A complex diagnosis such as COS would benefit from longitudinal follow-up to rule out confounding clinical presentations, particularly with regard to the influence of neurodevelopmental conditions on disorganized symptoms. Future work will be directed toward a longitudinal study of these patients, which remains challenging given the rarity and complexity of the disorder. Despite these limitations, the study provides novel insights into a rare and understudied population and underscores the need for further longitudinal research.

## 6 Conclusions

These findings suggest that COS may not be a homogeneous condition, but rather spans a range of psychopathological profiles that vary along a continuum, associated with different comorbidity patterns. Comorbidities are not merely a categorical overlap between disorders; rather, they may serve as clinically meaningful markers for navigating the complex psychopathological landscape of schizophrenia. In our sample, neurodevelopmental comorbidity was associated with a specific configuration of symptoms, characterized by earlier onset, lower IQ, greater bizarre thinking, and less social anhedonia. Recognizing these profiles may enhance early diagnostic characterization and support the development of targeted interventions. Given the substantial psychopathological continuity between COS, EOS, and AOS, advancing the clinical understanding of COS may contribute to improved outcomes across developmental stages.

## Data Availability

The raw data supporting the conclusions of this article will be made available by the authors, without undue reservation.
