# Supplementary material for: Rethinking childhood-onset schizophrenia: neurodevelopmental comorbidities shape the psychopathological profile
Source: Front Psychiatry. 2026 Oct 12;17:1918377. doi: 10.3389/fpsyt.2026.1918377 (PMC13696399; doi:10.3389/fpsyt.2026.1918377)
Supplement: Supplementary file 1 [file Table1.docx]

Supplementary Material

**Table S1. Descriptive characteristics of the nine participants excluded from the final sample.**

| **Patient** | **Reason for exclusion** |
| --- | --- |
| Patient 1 | Comorbid epilepsy |
| Patient 2 | Diagnosis of encephalitis |
| Patient 3 | Previous brain trauma |
| Patient 4 | Medication-induced psychotic disorder (high-dose venlafaxine) |
| Patient 5 | ASD + panic disorder |
| Patient 6 | ASD + OCD |
| Patient 7 | ID + depression |
| Patient 8 | ID + ADHD + social anxiety |
| Patient 9 | ADHD + ASD + OCD |

*Abbreviations: ID, intellectual disability; ADHD, attention-deficit/hyperactivity disorder; ASD, autism spectrum disorder; OCD, obsessive-compulsive disorder.*

*Note: Each participant is reported anonymously, together with the specific comorbidity or exclusion criterion that determined their exclusion from the final sample.*

**Table S2. Sex differences in SIPS/SOPS symptom dimensions.**

| **Variables** | **Males** | **Females** | **F** | **p-value** |  |
| --- | --- | --- | --- | --- | --- |
| ***SIPS Positive items*** |  |  |  |  |  |
| P1: Unusual Thought Content/Delusional Ideas | 4.86 (1.97) | 4.81 (1.85) | 0.01 | 0.921 |  |
| P2: Suspiciousness/Persecutory Ideas | 3.89 (2.18) | 3.92 (2.27) | 0.004 | 0.948 |  |
| P3: Grandiose Ideas | 1.40 (1.06) | 1.77 (1.47) | 1.29 | 0.261 |  |
| P4: Perceptual Abnormalities/Hallucinations | 3.91 (2.45) | 4.38 (2.22) | 0.59 | 0.445 |  |
| P5: Disorganized Communication | 2.57 (2.18) | 2.54 (1.94) | 0.004 | 0.952 |  |
| P total | 16.63 (5.06) | 17.42 (4.51) | 0.40 | 0.528 |  |
| ***SIPS Negative items*** |  |  |  |  |  |
| N1: Social Anhedonia | 5.03 (1.56) | 5.50 (1.27) | 1.58 | 0.213 | |
| N2: Avolition | 5.26 (1.40) | 4.62 (1.87) | 2.34 | 0.131 | |
| N3: Expression of Emotion | 5.23 (1.31) | 5.00 (1.60) | 0.37 | 0.542 | |
| N4: Experience of Emotions and Self | 5.17 (1.32) | 4.96 (1.64) | 0.31 | 0.581 | |
| N5: Ideational Richness | 4.63 (1.72) | 4.54 (1.79) | 0.04 | 0.843 | |
| N6: Occupational Functioning | 4.86 (1.31) | 4.62 (1.52) | 0.44 | 0.509 | |
| N total | 30.17 (6.27) | 29.23 (7.40) | 0.28 | 0.593 | |
| ***SIPS Disorganized items*** |  |  |  |  | |
| D1: Odd Behavior of Appearance | 5.43 (0.97) | 4.19 (1.98) | 3.00 | 0.088 | |
| D2: Bizarre Thinking | 5.14 (1.14) | 4.19 (1.98) | 5.58 | 0.021* | |
| D3: Trouble with Focus and Attention | 4.97 (1.74) | 3.42 (1.96) | 10.58 | 0.002* | |
| D4: Impairment in Personal Hygiene | 3.54 (1.94) | 2.81 (1.69) | 2.37 | 0.129 | |
| D total | 19.09 (3.91) | 15.19 (6.41) | 8.61 | 0.005* | |

* p ≤ 0.05

*Abbreviations:* SIPS: Structured Interview for Psychosis-Risk Syndrome

**Table S3. SIPS items in the total sample and comparisons across the three COS groups adjusting for IQ.**

|  | F | p |
| --- | --- | --- |
| *SIPS Positive items* |  |  |
| P1: Unusual Thought Content/Delusional Ideas | 1.646 | .189 |
| P2: Suspiciousness/Persecutory Ideas | 0.199 | .897 |
| P3: Grandiose Ideas | 0.315 | .814 |
| P4: Perceptual Abnormalities/Hallucinations | 0.916 | .439 |
| P5: Disorganized Communication | 0.437 | .727 |
| P total | 0.031 | .992 |
| *SIPS Negative items* |  |  |
| **N1: Social Anhedonia** | **5.831** | **.002** |
| N2: Avolition | 1.475 | .231 |
| N3: Expression of Emotion | 0.689 | .563 |
| N4: Experience of Emotions and Self | 0.846 | .474 |
| N5: Ideational Richness | 3.301 | .027 |
| N6: Occupational Functioning | 1.504 | .223 |
| N total | 2.919 | .042 |
| *SIPS Disorganized items* |  |  |
| D1: Odd Behavior or Appearance | 1.250 | .300 |
| **D2: Bizarre Thinking** | **4.841** | **.005** |
| D3: Trouble with Focus and Attention | 0.482 | .696 |
| D4: Impairment in Personal Hygiene | 0.998 | .401 |
| D total | 1.899 | .140 |
